# 4-Chloro-2-[(*E*)-(4-fluoro­phen­yl)imino­meth­yl]phenol

**DOI:** 10.1107/S1600536813033278

**Published:** 2013-12-14

**Authors:** Tian-Jun Feng

**Affiliations:** a College of Mathematics and Physics, Lanzhou Jiaotong University, Lanzhou 730070, People’s Republic of China

## Abstract

In the title Schiff base mol­ecule, C_13_H_9_ClFNO, the benzene rings are twisted slightly with respect to each other, making a dihedral angle of 7.92 (2)°. An intra­molecular O—H⋯N hydrogen bond occurs. In the crystal, an infinite chain is formed along the *c*-axis direction by π–π stacking inter­actions between the phenyl rings and the six-membered hydrogen-bonded ring of neighboring Schiff base ligands [centroid–centroid distances of 3.698 (2) and 3.660 (3) Å]. Neighboring chains are linked into a three-dimensional supra­molecular structure by C—H⋯O and C—H⋯F hydrogen bonds.

## Related literature   

For the coordination modes of Schiff base ligands with transition metals, see: Ebrahimipour *et al.* (2012 ▶); Guo *et al.* (2013 ▶). For the biological activity of Schiff base ligands, see: Sawada *et al.* (2001 ▶); Ma *et al.* (2013 ▶); Siddiqui *et al.* (2006 ▶).
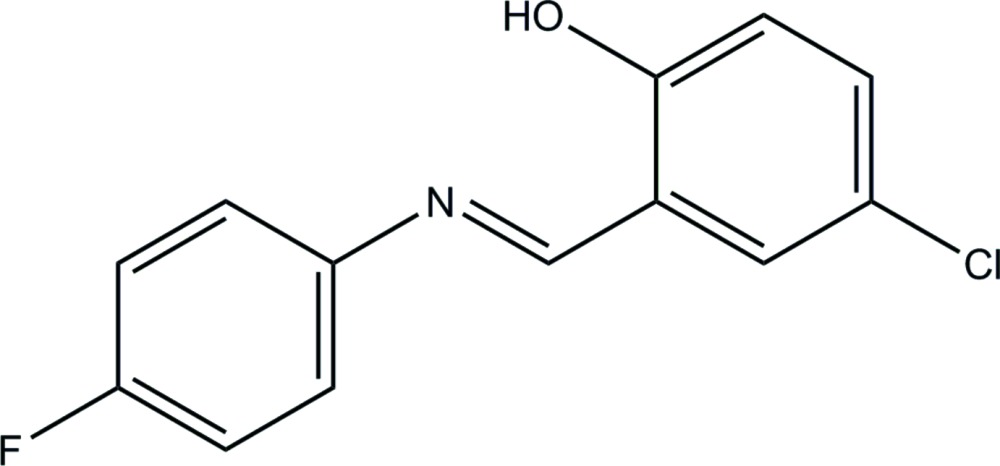


## Experimental   

### 

#### Crystal data  

C_13_H_9_ClFNO
*M* *_r_* = 249.66
Monoclinic, 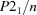

*a* = 4.5140 (9) Å
*b* = 20.560 (4) Å
*c* = 12.0712 (19) Å
β = 94.153 (16)°
*V* = 1117.4 (3) Å^3^
*Z* = 4
Mo *K*α radiation
μ = 0.34 mm^−1^
*T* = 293 K
0.34 × 0.27 × 0.22 mm

#### Data collection  

Agilent Xcalibur (Eos, Gemini) diffractometer
Absorption correction: multi-scan (*CrysAlis PRO*; Agilent, 2011 ▶) *T*_min_ = 0.908, *T*_max_ = 0.942
6481 measured reflections
2016 independent reflections
1143 reflections with *I* > 2σ(*I*)
*R*_int_ = 0.065

#### Refinement  

*R*[*F* ^2^ > 2σ(*F* ^2^)] = 0.056
*wR*(*F* ^2^) = 0.153
*S* = 1.04
2016 reflections
155 parameters
H-atom parameters constrained
Δρ_max_ = 0.19 e Å^−3^
Δρ_min_ = −0.23 e Å^−3^

### 

Data collection: *CrysAlis PRO* (Agilent, 2011 ▶); cell refinement: *CrysAlis PRO*; data reduction: *CrysAlis PRO*; program(s) used to solve structure: *SHELXS97* (Sheldrick, 2008 ▶); program(s) used to refine structure: *SHELXL97* (Sheldrick, 2008 ▶); molecular graphics: *ORTEPIII* (Burnett & Johnson, 1996 ▶); software used to prepare material for publication: *SHELXL97*.

## Supplementary Material

Crystal structure: contains datablock(s) I. DOI: 10.1107/S1600536813033278/zl2572sup1.cif

Structure factors: contains datablock(s) I. DOI: 10.1107/S1600536813033278/zl2572Isup2.hkl

Supporting information file. DOI: 10.1107/S1600536813033278/zl2572Isup3.cml

Additional supporting information:  crystallographic information; 3D view; checkCIF report

## Figures and Tables

**Table 1:** Hydrogen-bond geometry (Å, °)

*D*—H⋯*A*	*D*—H	H⋯*A*	*D*⋯*A*	*D*—H⋯*A*
C7—H7⋯O1^i^	0.93	2.69	3.569 (4)	158
C10—H10⋯F1^ii^	0.93	2.67	3.481 (4)	147
O1—H1⋯N1	0.82	1.88	2.613 (3)	148

Symmetry codes: (i) 
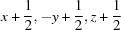
; (ii) 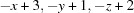.

**Fig. 1.**
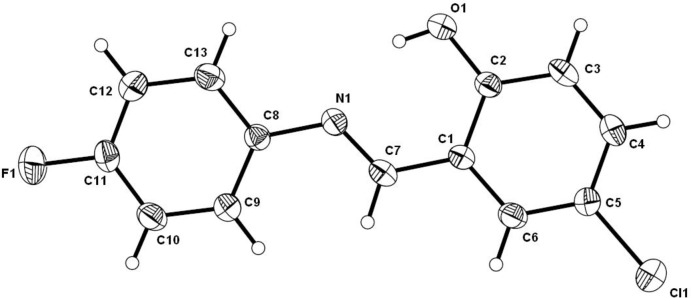
The molecular structure of the title compound, showing 30% probability displacement ellipsoids.

**Fig. 2.**
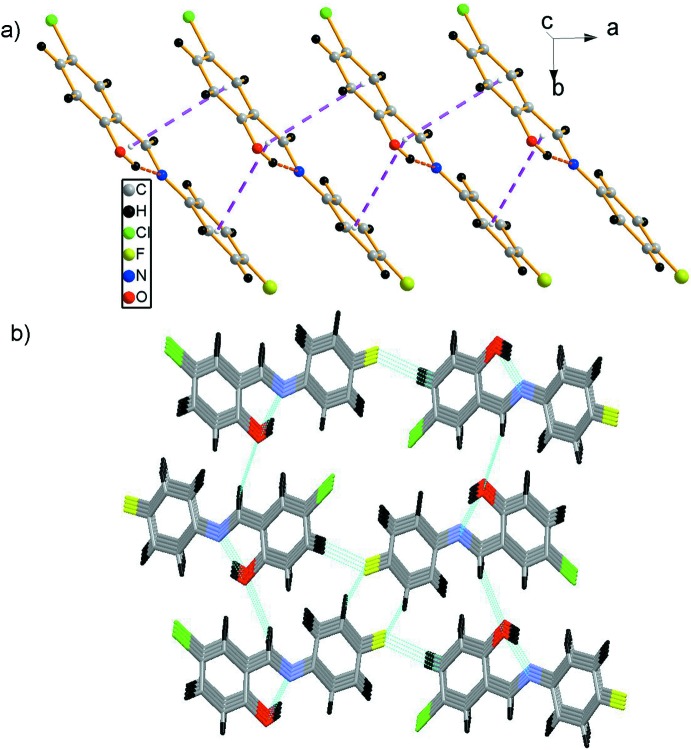
(a) View of an infinite chain of the title compound formed by π-π stacking interactions (purple dashed lines); (b) View of the three-dimensional supramolecular structure of the title compound formed by C—H···O and C—H···F hydrogen bonds (blue dashed lines).

## supplementary crystallographic information

### 1. Comment

Schiff bases are considered important compounds because of their wide range of biological activities, and also because of their use as ligands in conjunction with transition metals. Schiff base ligands usually coordinate to a metal ion through the imine nitrogen atom, but coordination *via* other functional groups, *e.g.* through oxygen or carbon, has also been reported (Ebrahimipour *et al.*, 2012; Guo *et al.*, 2013). Schiff bases derived from salicyladehyde and fluoroaniline, specifically, have been considered as potential pharmaceutically interesting compounds as several of the members of this family of molecules have shown antitumor, antimicrobial or antiviral activities (Sawada *et al.*, 2001; Ma *et al.*, 2013; Siddiqui *et al.*, 2006). As an extension of our work on the structural characterization of Schiff base compounds, the solid state structure of the title compound is reported.

The molecular structure of the title compound shows an E configuration, with a C8—N1=C7—C1 torsion angle of 178.33 (2) °. The bond distance of N1=C7 at 1.276 (3) Å is a typical double bond. It is noteworthy that the H1 atom bonded to O1 is involved in an O1—H1···N1 intramolecular hydrogen bond, which results in the formation of a six-membered ring (Table 1). The dihedral angle between the two planes of the chlorophenol ring and fluorphenyl ring is 7.92 (2) °. An infinite chain is formed by two types of π-π stacking interactions between the phenyl rings (C1—C6 and C8—C13) and the six-membered hydrogen bonded ring (C1/C2/O1/H1/N1/C7) of neighboring Schiff base ligands, with centroid–centroid distances of 3.698 (2) and 3.660 (3) Å, respectively and interplanar spacings of 3.395 (2) Å (Fig. 2a). Finally, neighboring chains are linked into a three-dimensional supramolecular structure by weak C—H···O and C—H···F hydrogen bonding interactions (Fig. 2 b, Table 1).

### 2. Experimental

Title compound was prepared by the condensation of 5-chlorosalicylaldehyde (0.783 g, 5 mmol) and 4-fluoroaniline (0.556 g, 5 mmol) in ethanol (15 ml) as the reaction medium. Glacial acetic acid (0.4 ml) was added and the solution was heated under reflux for 5 h and then allowed to cool to room temperature. The yellow precipitate was recrystallized from ethanol to give the title compound as straw yellow crystals. Yield 0.20 g (80%). [m.p. 361–363 K; IR (KBr, cm^-1^): 1637(s), 1560(m), 1508(w), 1460(w), 1392(w), 1324(w), 1288(w), 1210(w), 1120(w), 1054(w), 982(w), 932(w), 876(w), 810(w), 747(w), 709(w), 675(w), 564(w), 511(w); ^1^H NMR (CDCl_3_, δ, p.p.m.) 13.11 (s, 1H), 8.55 (s, 1H), 6.99–7.39 (m, 7H); ^13^C NMR (CDCl_3_, δ, p.p.m.) 161.1, 161.0, 160.7, 159.6, 144.2, 144.1, 133.0, 131.2, 123.8, 122.7, 122.6, 119.9, 118.9, 116.5, 116.2].

### 3. Refinement

H atoms were fixed geometrically and treated as riding with O—H = 0.82 Å (hydroxy) and C—H = 0.93 Å, *U*_iso_(H) = 1.2 *U*_eq_(C) for aromatic H atoms and *U*_iso_(H) = 1.5 *U*_eq_(O) for the hydroxy H atom. The hightest residual electron density peak is located 0.91 Å from H1 and the deepest hole is located 0.91 Å from C13.

### Crystal data

**Table:**

C_13_H_9_ClFNO	*F*(000) = 512
*M**_r_* = 249.66	*D*_x_ = 1.484 Mg m^−^^3^
Monoclinic, *P*2_1_/*n*	Mo *K*α radiation, λ = 0.71073 Å
Hall symbol: -P 2yn	Cell parameters from 3100 reflections
*a* = 4.5140 (9) Å	θ = 1.7–26.0°
*b* = 20.560 (4) Å	µ = 0.34 mm^−^^1^
*c* = 12.0712 (19) Å	*T* = 293 K
β = 94.153 (16)°	Block, yellow
*V* = 1117.4 (3) Å^3^	0.34 × 0.27 × 0.22 mm
*Z* = 4	

### Data collection

**Table:**

Agilent Xcalibur (Eos, Gemini) diffractometer	2016 independent reflections
Radiation source: fine-focus sealed tube	1143 reflections with *I* > 2σ(*I*)
Graphite monochromator	*R*_int_ = 0.065
ω scans	θ_max_ = 25.2°, θ_min_ = 2.6°
Absorption correction: multi-scan (*CrysAlis PRO*; Agilent, 2011)	*h* = −5→5
*T*_min_ = 0.908, *T*_max_ = 0.942	*k* = −23→24
6481 measured reflections	*l* = −14→14

### Refinement

**Table:**

Refinement on *F*^2^	Primary atom site location: structure-invariant direct methods
Least-squares matrix: full	Secondary atom site location: difference Fourier map
*R*[*F*^2^ > 2σ(*F*^2^)] = 0.056	Hydrogen site location: inferred from neighbouring sites
*wR*(*F*^2^) = 0.153	H-atom parameters constrained
*S* = 1.04	*w* = 1/[σ^2^(*F*_o_^2^) + (0.0516*P*)^2^] where *P* = (*F*_o_^2^ + 2*F*_c_^2^)/3
2016 reflections	(Δ/σ)_max_ < 0.001
155 parameters	Δρ_max_ = 0.19 e Å^−^^3^
0 restraints	Δρ_min_ = −0.23 e Å^−^^3^

### Special details

**Table:**

Geometry. All esds (except the esd in the dihedral angle between two l.s. planes) are estimated using the full covariance matrix. The cell esds are taken into account individually in the estimation of esds in distances, angles and torsion angles; correlations between esds in cell parameters are only used when they are defined by crystal symmetry. An approximate (isotropic) treatment of cell esds is used for estimating esds involving l.s. planes.
Refinement. Refinement of F^2^ against ALL reflections. The weighted R-factor wR and goodness of fit S are based on F^2^, conventional R-factors R are based on F, with F set to zero for negative F^2^. The threshold expression of F^2^ > 2sigma(F^2^) is used only for calculating R-factors(gt) etc. and is not relevant to the choice of reflections for refinement. R-factors based on F^2^ are statistically about twice as large as those based on F, and R- factors based on ALL data will be even larger.

### Fractional atomic coordinates and isotropic or equivalent isotropic displacement parameters (Å^2^)

**Table:**

	*x*	*y*	*z*	*U*_iso_*/*U*_eq_	
C1	0.2601 (7)	0.22941 (16)	0.7977 (3)	0.0364 (8)	
C2	0.1287 (7)	0.22602 (17)	0.6887 (3)	0.0409 (8)	
C3	−0.0847 (8)	0.17908 (18)	0.6617 (3)	0.0485 (10)	
H3	−0.1728	0.1772	0.5897	0.058*	
C4	−0.1685 (8)	0.13523 (18)	0.7395 (3)	0.0480 (9)	
H4	−0.3112	0.1038	0.7202	0.058*	
C5	−0.0384 (8)	0.13834 (17)	0.8465 (3)	0.0462 (9)	
C6	0.1714 (7)	0.18464 (16)	0.8761 (3)	0.0456 (9)	
H6	0.2552	0.1863	0.9488	0.055*	
C7	0.4848 (7)	0.27750 (17)	0.8303 (3)	0.0420 (9)	
H7	0.5699	0.2772	0.9028	0.050*	
C8	0.7834 (7)	0.36833 (16)	0.7949 (3)	0.0380 (8)	
C9	0.8940 (7)	0.37974 (17)	0.9038 (3)	0.0477 (9)	
H9	0.8310	0.3540	0.9610	0.057*	
C10	1.0968 (8)	0.42908 (17)	0.9277 (3)	0.0517 (10)	
H10	1.1698	0.4370	1.0005	0.062*	
C11	1.1880 (8)	0.46601 (17)	0.8424 (3)	0.0487 (9)	
C12	1.0866 (8)	0.45657 (18)	0.7349 (3)	0.0524 (10)	
H12	1.1526	0.4824	0.6785	0.063*	
C13	0.8819 (8)	0.40728 (17)	0.7116 (3)	0.0494 (10)	
H13	0.8093	0.4002	0.6385	0.059*	
Cl1	−0.1447 (3)	0.08228 (5)	0.94489 (9)	0.0769 (4)	
F1	1.3866 (5)	0.51483 (10)	0.86620 (19)	0.0767 (7)	
N1	0.5694 (6)	0.32018 (13)	0.7626 (2)	0.0407 (7)	
O1	0.2061 (6)	0.26776 (13)	0.60954 (19)	0.0573 (7)	
H1	0.3290	0.2937	0.6366	0.086*	

### Atomic displacement parameters (Å^2^)

**Table:**

	*U*^11^	*U*^22^	*U*^33^	*U*^12^	*U*^13^	*U*^23^
C1	0.0361 (18)	0.039 (2)	0.0332 (19)	0.0018 (16)	−0.0011 (15)	−0.0019 (16)
C2	0.044 (2)	0.043 (2)	0.035 (2)	−0.0010 (17)	0.0013 (16)	−0.0047 (17)
C3	0.052 (2)	0.054 (2)	0.038 (2)	0.0012 (19)	−0.0047 (18)	−0.0142 (19)
C4	0.048 (2)	0.044 (2)	0.051 (2)	−0.0036 (18)	0.0013 (19)	−0.0134 (19)
C5	0.053 (2)	0.041 (2)	0.044 (2)	−0.0051 (18)	0.0023 (18)	−0.0012 (17)
C6	0.051 (2)	0.048 (2)	0.037 (2)	0.0005 (18)	−0.0034 (17)	−0.0033 (18)
C7	0.0398 (19)	0.046 (2)	0.039 (2)	0.0023 (17)	−0.0052 (16)	−0.0055 (18)
C8	0.0378 (19)	0.036 (2)	0.039 (2)	0.0013 (15)	−0.0008 (16)	−0.0005 (17)
C9	0.052 (2)	0.048 (2)	0.043 (2)	−0.0075 (18)	0.0015 (18)	−0.0019 (18)
C10	0.059 (2)	0.049 (2)	0.045 (2)	−0.0026 (19)	−0.0100 (19)	−0.0030 (19)
C11	0.045 (2)	0.042 (2)	0.058 (3)	−0.0080 (17)	−0.0024 (19)	−0.004 (2)
C12	0.056 (2)	0.049 (2)	0.053 (2)	−0.005 (2)	0.007 (2)	0.0058 (19)
C13	0.052 (2)	0.053 (2)	0.042 (2)	0.0011 (19)	−0.0058 (18)	0.0001 (19)
Cl1	0.0965 (9)	0.0668 (8)	0.0659 (7)	−0.0274 (6)	−0.0031 (6)	0.0137 (6)
F1	0.0852 (16)	0.0563 (15)	0.0870 (17)	−0.0318 (13)	−0.0043 (13)	−0.0019 (13)
N1	0.0397 (16)	0.0414 (17)	0.0407 (17)	−0.0025 (14)	0.0010 (13)	−0.0040 (15)
O1	0.0689 (19)	0.0628 (18)	0.0385 (14)	−0.0163 (14)	−0.0068 (13)	0.0018 (14)

### Geometric parameters (Å, º)

**Table:**

C1—C6	1.400 (4)	C8—C13	1.384 (4)
C1—C2	1.406 (4)	C8—C9	1.392 (4)
C1—C7	1.450 (4)	C8—N1	1.418 (4)
C2—O1	1.349 (4)	C9—C10	1.383 (5)
C2—C3	1.385 (4)	C9—H9	0.9300
C3—C4	1.374 (5)	C10—C11	1.366 (5)
C3—H3	0.9300	C10—H10	0.9300
C4—C5	1.381 (5)	C11—C12	1.359 (5)
C4—H4	0.9300	C11—F1	1.362 (4)
C5—C6	1.372 (4)	C12—C13	1.386 (5)
C5—Cl1	1.747 (4)	C12—H12	0.9300
C6—H6	0.9300	C13—H13	0.9300
C7—N1	1.276 (4)	O1—H1	0.8200
C7—H7	0.9300		
			
C6—C1—C2	118.6 (3)	C13—C8—C9	118.5 (3)
C6—C1—C7	119.6 (3)	C13—C8—N1	116.9 (3)
C2—C1—C7	121.8 (3)	C9—C8—N1	124.6 (3)
O1—C2—C3	119.2 (3)	C10—C9—C8	120.5 (3)
O1—C2—C1	121.2 (3)	C10—C9—H9	119.8
C3—C2—C1	119.5 (3)	C8—C9—H9	119.8
C4—C3—C2	121.2 (3)	C11—C10—C9	118.8 (3)
C4—C3—H3	119.4	C11—C10—H10	120.6
C2—C3—H3	119.4	C9—C10—H10	120.6
C3—C4—C5	119.3 (3)	C12—C11—F1	118.5 (3)
C3—C4—H4	120.4	C12—C11—C10	122.8 (4)
C5—C4—H4	120.4	F1—C11—C10	118.7 (3)
C6—C5—C4	121.0 (3)	C11—C12—C13	118.1 (4)
C6—C5—Cl1	119.9 (3)	C11—C12—H12	120.9
C4—C5—Cl1	119.1 (3)	C13—C12—H12	120.9
C5—C6—C1	120.4 (3)	C8—C13—C12	121.3 (3)
C5—C6—H6	119.8	C8—C13—H13	119.3
C1—C6—H6	119.8	C12—C13—H13	119.3
N1—C7—C1	122.1 (3)	C7—N1—C8	122.3 (3)
N1—C7—H7	119.0	C2—O1—H1	109.5
C1—C7—H7	119.0		

### Hydrogen-bond geometry (Å, º)

**Table:**

*D*—H···*A*	*D*—H	H···*A*	*D*···*A*	*D*—H···*A*
C7—H7···O1^i^	0.93	2.69	3.569 (4)	158
C10—H10···F1^ii^	0.93	2.67	3.481 (4)	147
O1—H1···N1	0.82	1.88	2.613 (3)	148

Symmetry codes: (i) *x*+1/2, −*y*+1/2, *z*+1/2; (ii) −*x*+3, −*y*+1, −*z*+2.
